# Mapping the exposome of Zimbabwean preschool-aged children using a novel peptide microarray approach

**DOI:** 10.3389/fimmu.2026.1915960

**Published:** 2026-10-01

**Authors:** Emilee J. Benos, Lorraine T. Pfavayi, Derick N.M. Osakunor, Takafira Mduluza, Francisca Mutapi

**Affiliations:** 1 Ashworth Laboratories, Institute of Immunology and Infection Research, Department of Biological Sciences, University of Edinburgh, Edinburgh, United Kingdom; 2 Tackling Infections to Benefit Africa (TIBA) Partnership, Ashworth Laboratories, The University of Edinburgh, Edinburgh, United Kingdom; 3 School of Biodiversity, One Health and Veterinary Medicine, University of Glasgow, Glasgow, United Kingdom; 4 Department of Biochemistry, University of Zimbabwe, Harare, Zimbabwe

**Keywords:** antibody, exposome, immune variation, immunity, peptide microarray

## Abstract

Immune responses vary substantially between individuals and populations, reflecting differences in genetics, environment, and cumulative antigenic exposures. Characterizing these exposures, or the immunological exposome, is essential for understanding population-specific immune development, susceptibility to infection, and responses to interventions. Here, we present a proof-of-concept study applying a bespoke high-density peptide microarray to map the exposome of Zimbabwean preschool-aged children (PSAC). Using serum samples from 20 children under five years of age living in a schistosomiasis-endemic area, we profiled IgM and IgG responses against 5,614 peptides representing 280 pathogens, vaccines, allergens, and autoantigens. As internal validation, we used childhood vaccine antigens which the clinical records indicated the children had received. The platform successfully detected antibody responses to all the childhood vaccine antigens represented on the array, demonstrating sensitivities ranging from 87.5–100% for IgM and 61.5–100% for IgG. Beyond vaccine-associated responses, children exhibited diverse and highly heterogeneous antibody profiles, with strong reactivity to enteroviruses, *Streptococcus* spp., *S, pneumoniae*, *Clostridium perfringens*, *Clostridioides difficile*, and the zoonotic pathogen *Ehrlichia canis*. Distinct IgM and IgG signatures revealed evidence of both recent and historical antigenic exposures, highlighting the breadth of immune stimulation experienced early in life. These findings demonstrate the feasibility of high-throughput serological exposome mapping in African children and provide the first detailed characterization of the childhood immunological exposome in this setting. This approach offers a powerful tool for investigating immune heterogeneity, informing vaccine development, and establishing population-specific immune reference frameworks in high-burden environments.

## 1 Introduction

Immune function varies considerably between individuals and populations, and is driven by factors including genetics, age, environment, and previous and concurrent infections (1–3). This heterogeneity contributes to population-level differences in susceptibility to pathogens, disease incidence and severity, and therapeutic responses.

For example, numerous studies show that rural African populations exhibit distinct immune profiles and responses to infections and interventions compared with their Western counterparts. Evidence shows differences in the percentage of immune cell subsets in sub-Saharan African individuals compared to individuals from other regions (4). Other studies have reported lower leukocyte and neutrophil counts in African individuals compared to those from the United States (5), as well as low CD4+ T cell counts (6, 7), and activated immune profiles in otherwise healthy African individuals (8, 9). Such immunological patterns may contribute to differences in infectious disease outcomes across regions. For example, The SARS-CoV-2 pandemic highlighted population level immune differences, with many African countries reporting lower infection and mortality rates compared to other continents (10–13). Additionally, evidence shows that vaccine efficacy rates are often lower in African countries than in Western countries (9, 14–21).

Further, there is evidence that immune system variations can be found not only at the continental level, but also within countries in Africa. Research has shown that country-specific variations in immune system profiles are present from early life, and are linked to differential responses to the malaria vaccine. For example, children in Mozambique have been shown to exhibit a more activated baseline immune profile, with higher frequencies of circulating T follicular helper and antibody-secreting cells, which were associated with higher IgG responses to the RTS,S malaria vaccine compared with children in Tanzania (3). These findings highlight the importance of incorporating population-specific immunological data into public health planning strategies.

The first five years of life represent a critical window for immune system development and calibration, during which repeated exposures to pathogens and vaccines shape both the regulatory and memory arms of adaptive immunity (22, 23). This period is characterized by evolving quantitative and qualitative differences in humoral and cellular responses compared to adults (22). These developmental differences include delayed germinal center formation and altered T-B cell interactions that shape early life IgM and IgG responses (24). Further, diverse early-life exposures have implications for immune regulation and disease susceptibility, as early antigenic contacts help educate the immune system and may establish immunological memory that influences later responses (23, 25). Previous age-stratified seroepidemiological studies from low-resource, high-burden settings confirm that exposure to many pathogens occurs within the first few years of life, with IgG seroconversion occurring before age 3 for numerous endemic agents (26–29). Early infections and antigenic exposures profoundly influence immune regulation, with mechanisms such as Th1/Th2 balance and innate-like adaptive responses evolving in response to environmental stimuli (30, 31). This period is also characterized by distinct features of early adaptive immunity, including a high reliance on innate-like effector function and a developing capacity for durable memory formation, which together determine the magnitude, quality, and longevity of antibody and cellular responses elicited by both natural infection and vaccination (31). Moreover, early antigenic priming can generate cross-reactive antibody and T cell repertoires that confer partial protection against related pathogens and influence vaccine efficacy. This effect is especially relevant in settings of high microbial diversity and repeated exposures (32). Understanding this combined influence of early pathogen and vaccine-driven priming on immune phenotype in this age range can improve insight on immune trajectories that may impact health later in life.

Characterizing heterogeneity at the individual level is essential before drawing conclusions about population differences. Variation in exposure history, infection timing, vaccine uptake, genetics, nutrition, and microbiome composition can produce markedly different immune phenotypes within the same community. Without first characterizing this within-population variability, between-population comparisons risk oversimplification or ecological fallacy, obscuring important subgroups and misattributing differences to geography or ethnicity rather than underlying exposure or immunological factors. Therefore, careful analysis of individual-level heterogeneity provides the foundation for interpreting broader population contrasts in a biologically meaningful way.

One approach to characterizing individual immune responses within a population is through serological surveys. Antibodies are a strong correlate of recent exposure to immunogens and protective immunity for many diseases (33). The quantification of antibody titers therefore provides valuable insights on a population’s exposure history and humoral immune signatures, as serological profiling captures antigenic experience and reflects both infection- and vaccine-induced immune memory (34). Understanding a population’s exposome, defined as the collection of an individual’s exposures to immunogens (35), via serological studies can reveal important immunological heterogeneities and help to define how host antigenic experiences shape the immune landscape. Early childhood is a period of rapid immune maturation, during which exposures can exert lasting effects on immune architecture, growth, vaccine responsiveness, infection susceptibility, and risk of immune-mediated and non-communicable diseases. Mapping which epitopes are recognized early in life across pathogens, allergens, vaccines, and autoantigens is critical for understanding how population-specific immune profiles develop.

Despite growing recognition of the importance of the exposome in shaping immune function, population-level data from African children remain scarce. We therefore used a bespoke high-density peptide microarray to map the immunological exposome of Zimbabwean preschool-aged children. By simultaneously profiling IgM and IgG responses to a broad panel of pathogen-, vaccine-, allergen-, and autoantigen-derived epitopes, we sought to determine whether multiplex serological profiling could capture the breadth and heterogeneity of early-life antigenic exposures. Specifically, this proof-of-concept study aimed to establish whether such an approach could define population-specific immune landscapes and provide a foundation for exposome-informed investigations of immune development, vaccine responsiveness, and population health in African settings.

## 2 Materials and methods

### 2.1 Ethical approval and consent

The current study forms part of a broader study aimed at profiling the immunological and molecular phenotypes of schistosome-endemic human responses. Ethical and institutional approval for this study was obtained from the Medical Research Council of Zimbabwe (MRCZ/E/167) and the University of Edinburgh (fmutapi-0002). The Mashonaland Central Provincial Medical Director granted permission to conduct the study. Study procedures and objectives were explained to participants and their parents and/or guardians in their local language, Shona, prior to enrolment. Participants’ parents/guardians provided written informed consent. Recruitment in the study was voluntary and participants were free to withdraw at any stage. Only samples from consenting participants were included in the current sub-study.

### 2.2 Study design, population, and site

The serum samples and corresponding metadata analyzed in this study represent a subset (n = 20) selected from a cohort of Zimbabwean preschool-aged children (PSAC; 6 months to 5 years) participating in a larger longitudinal study comparing treatment regimens for pediatric schistosomiasis (36). These samples were collected from a *S. haematobium*-endemic region in the Shamva district, Northeast Zimbabwe, between February 2016 and February 2017. Briefly, children in this age group with no history of malaria/fever, tuberculosis, or recent major illness or surgery and who were lifelong residents of the area were included in the main baseline survey. The participants were screened for *S. haematobium* via parasitological diagnosis at baseline. Samples used in this study comprised both *S. haematobium*-positive and -negative children from the baseline survey (n = 10 per group). This balanced selection was intended to ensure representation of both infection states within the small proof-of-concept sample and to capture heterogeneity in endemic exposure backgrounds, rather than to constitute a powered case-control comparison of antibody responses according to schistosomiasis status. At the time of recruitment, a questionnaire was administered to gather metadata on socio-demography and clinical history. Participants were eligible for inclusion if they had completed parasitological testing via urine filtration on three consecutive days (37) and accompanying study questionnaires. Participants were excluded if they had a reported lifetime history of malaria, febrile illness, tuberculosis, major illness, or any surgery. In terms of study design, we used the childhood vaccination and schistosomiasis seroconversion status/reactivity as assay positive controls as we had the clinical records confirming vaccination. As this study was designed as a proof-of-concept evaluation of the peptide microarray approach rather than a confirmatory hypothesis-testing study, a formal *a priori* sample size calculation was not performed. Twenty participants were included to enable an initial assessment of the breadth and heterogeneity of antibody reactivity and to generate preliminary findings for validation in larger cohorts.

### 2.3 Sample collection and processing

Briefly, 5mL of venous blood was collected in serum separator tubes (BD Vacutainer^®^) in the field, left to clot for 30 minutes at room temperature and centrifuged to separate serum. Serum was stored at -20 °C in the field, then transported to the University of Edinburgh where it was stored at -80 °C until used.

### 2.4 Peptide selection and microarray chip design

The high spot density (1000 peptides/cm2) of peptide microarrays makes them an efficient and cost-effective method for large scale serological surveys to generate a comprehensive fingerprint of a population’s systemic antibody responses. The bespoke PEPperCHIP^®^ TIBA microarrays used in this study included peptides from 4,405 B-cell epitopes identified from the Immune Epitope Database (IEDB) representative of immunogens endemic to Zimbabwe. A further 626 peptides from a previous commercially-available epitope discovery screening of very rare pathogen antigens were also included on the chip (38). Pathogen proteins that elicited the highest antibody responses from this epitope discovery screening were included on the final peptide microarray.

Overall, 280 immunogens were represented on the microarray. The peptides included on the microarray were derived from at least 2 different proteins from each pathogen to avoid overrepresentation and potential cross-reactivity. These immunogenic peptides were also screened for potential cross-reactivity using the NCBI Protein BLAST bioinformatics tool (https://blast.ncbi.nlm.nih.gov/Blast.cgi select Protein BLAST). The peptides that were the least cross-reactive with peptides from other human proteins or pathogens were selected for use in the study.

Multiple peptides were included for some antigens, reaching a final 5,614 peptides on the microarray. These identified peptides ranged 5–18 amino acids in length and were represented as overlapping 15-mer peptides. They were laser-printed in duplicate by PEPperPRINT GmbH (Heidelberg, Germany) (https://www.pepperprint.com/) onto the microarray, resulting in 11,228 spots total. These were representative of 280 immunogens including pathogens, allergens, autoantigens, and vaccines endemic to Zimbabwe, along with some less common antigens as negative controls. The microarray also included hemagglutinin (HA) and polio peptides as positive controls as all the children enrolled in the study had received polio vaccinations.

### 2.5 Antibody profiling

Antibody profiling was conducted by an industry specialist, PEPperPRINT GmbH (Heidelberg, Germany) following a previously published protocol (10). Briefly, the 15-mer microarray chip was screened with the serum samples we provided at 1:500 dilutions. PEPperCHIP^®^ TIBA Microarrays were initially incubated for 45 minutes at 37 °C with secondary antibodies (Goat anti-human IgG (Fc) DyLight680 (0.1 µg/ml) & goat anti-human IgM (µ chain) DyLight800 (0.2 µg/ml) to identify any background interactions that could potentially interfere with the main assays. To ensure assay performance and microarray integrity, internal quality control was performed by staining the HA control peptides along the frame of the microarray with a control antibody (mouse monoclonal anti-HA (12CA5) DyLight800 (0.2 µg/ml)). Rockland blocking buffer (MB-070) was used to detect signals of fluorescently labeled secondary antibodies with low background noise. This step blocked unwanted protein binding on the microarray, thereby reducing background signals and generating high-quality data. The PEPperCHIP^®^ TIBA Microarrays were subsequently incubated with the study serum samples at a 1:500 dilution for 16 hours at 4 °C, followed by secondary antibody staining. Results were read using an Innopsys InnoScan 710-IR Microarray Scanner and image analysis was performed with PepSlide^®^ Analyzer. The Analyzer has features that allow for both local and global background correction. We used a global background approach, where the region of the microarray showing no detectable signal was selected and defined in the software as the background. This background value was then subtracted from all measured signal intensities. Fluorescence intensities of each spot were broken down into raw, foreground, and background signals, and average median foreground intensities and spot-to-spot deviations (maximum of 40%) of spot duplicates were calculated via a MAPIX software algorithm. Fluorescent intensity maps were generated based on averaged median foreground intensities. An intensity color code with red (IgG) & green (IgM) for high and black for low spot intensities was used to highlight interactions in the peptide maps. IgM and IgG were selected as they are markers of recent/current antigen exposure (IgM) and memory responses (IgG). In addition to the internal assay controls, we included the polio vaccine peptide epitopes on the microarray chips as a positive control as all participants were confirmed recipients of the childhood polio vaccine.

### 2.6 Data analysis and visualization

GraphPad Prism 10.1 was used to analyze serological outputs. For descriptive analyses, antibody signals with an intensity greater than 0 were classified as exhibiting detectable reactivity, while signals of 0 were classified as non-reactive. This threshold was used as an operational definition to distinguish detectable from absent array signals and was not intended to represent a validated seropositivity cutoff. Primary analyses focused on the magnitude and relative ranking of antibody reactivities, particularly the highest-intensity responses, thereby limiting the influence of very low residual signals on the principal findings. Antibody reactivities were classed by immunogen of origin with the highest antibody intensity reading across peptides for IgM and IgG used for the heat map values. Immunogens that elicited an antibody response in at least half the study population were ordered by intensity levels and visualized using GraphPad Prism 10.1.1 to show the most relevant immunogens in the population.

### 2.7 Sensitivity calculation

Seroreactivity relative to documented vaccination history was evaluated separately for IgM and IgG. IgG was considered the more biologically appropriate marker of historical vaccine exposure because of its greater persistence following vaccination. IgM analyses were included on an exploratory basis to characterize detectable IgM reactivity within the broader serological profile and were not interpreted as a measure of long-term vaccine-induced immunity.

Sensitivities were calculated for vaccine scheduled pathogens to validate the microarray chip using the following equation:

Sensitivity (%) = [True Positive/(True Positive + False Negative)] × 100

## 3 Results

### 3.1 Participant characteristics

This study included 20 serum samples from children aged between 2 and 5 years. Males comprised 55% of the study population, while females accounted for 45%. Ten of the samples were negative for *S. haematobium*, and ten were positive based on parasitological diagnosis performed at the time of baseline collection. Samples included in the current study came from six villages across the Shamva district: Mupfure, Chihuri, Chakandora, Kaziro, and Nyakurunda, and Nduna (**Table 1**).

**Table 1:** Study population demography.

Sample size										
20 (100%)										
Sex										
Male					Female				
11 (55%)					9 (45%)				
Age										
1	2			3		4		5	
0 (0%)	4 (20%)			6 (30%)		8 (40%)		2 (10%)	
Village										
Chakandora		Chihuri	Kaziro		Mupfure		Nduna		Nyarukunda
1 (5%)		3 (15%)	1 (5%)		5 (25%)		8 (40%)		2 (10%)
Schistosomiasis Status										
Positive					Negative				
10 (50%)					10 (50%)				

### 3.2 Quality control and assay validation

Prestaining with secondary antibodies revealed a few weak background interactions with secondary goat anti-human IgM and a few very weak background interactions with secondary goat anti-human IgG. These background interactions were with basic peptides and likely due to non-specific ionic antibody binding. The HA control spots framing the microarray border appeared as expected following staining with both control antibodies, validating the microarray’s overall integrity and assay performance. Sensitivity for polio IgM and IgG was 100% and 85.7%, respectively.

### 3.3 Microarray chip validation

To validate assay performance, the microarray included antigens from routine childhood vaccines for which participants’ vaccination records were available. We therefore first assessed IgM and IgG responses to vaccine-associated antigens in the children to determine whether the platform could reliably detect known antigen exposures and corresponding serological signatures. IgM sensitivities for complete vaccination ranged from 87.5% (Haemophilus influenzae type B) to 100%. IgG sensitivities for complete vaccination ranged from 61.5% (measles, rubella) to 100% (**Table 2**). Rotavirus sensitivities could not be calculated as all participants remained unvaccinated against rotavirus. Contingency tables for sensitivity calculations can be found in **Supplementary Material 1**.

**Table 2:** Sensitivities of vaccine schedule pathogens compared to physical vaccination records.

Vaccine schedule pathogen	IgM Sensitivity	IgG Sensitivity
BCG	90.9%	90.9%
TB	100%	90.9%
Measles	100%	61.5%
Rubella	92.3%	61.5%
*B. pertussis*	100%	87.5%
*C. tetani*	100%	87.5%
*C. diphtheriae*	100%	87.5%
HiB	87.5%	87.5%
HepB	100%	87.5%
*S. pneumoniae*	100%	100%
Polio	100%	85.7%
Rotavirus	N/A	N/A
Meningitis	100%	100%

IgM responses were heterogeneous across individuals, with the strongest reactivities observed against poliovirus types 1 and 3, followed by *Bordetella pertussis*, *Clostridium tetani*, hepatitis B virus, and *Mycobacterium tuberculosis*. In contrast, rotavirus and measles antigens elicited the lowest IgM intensities. IgG responses exhibited a similar pattern, with poliovirus types 1 and 3 producing the highest reactivities across all vaccine antigens, followed by *Streptococcus pneumoniae*, *B. pertussis*, *M. tuberculosis*, and *C. tetani*. Lower IgG intensities were observed for Haemophilus influenzae type B, rotavirus, and rubella. (**Figures 1A, B**). For each vaccine, the IgM and IgG signals varied across all children for each peptide epitope.

**Figure 1:**
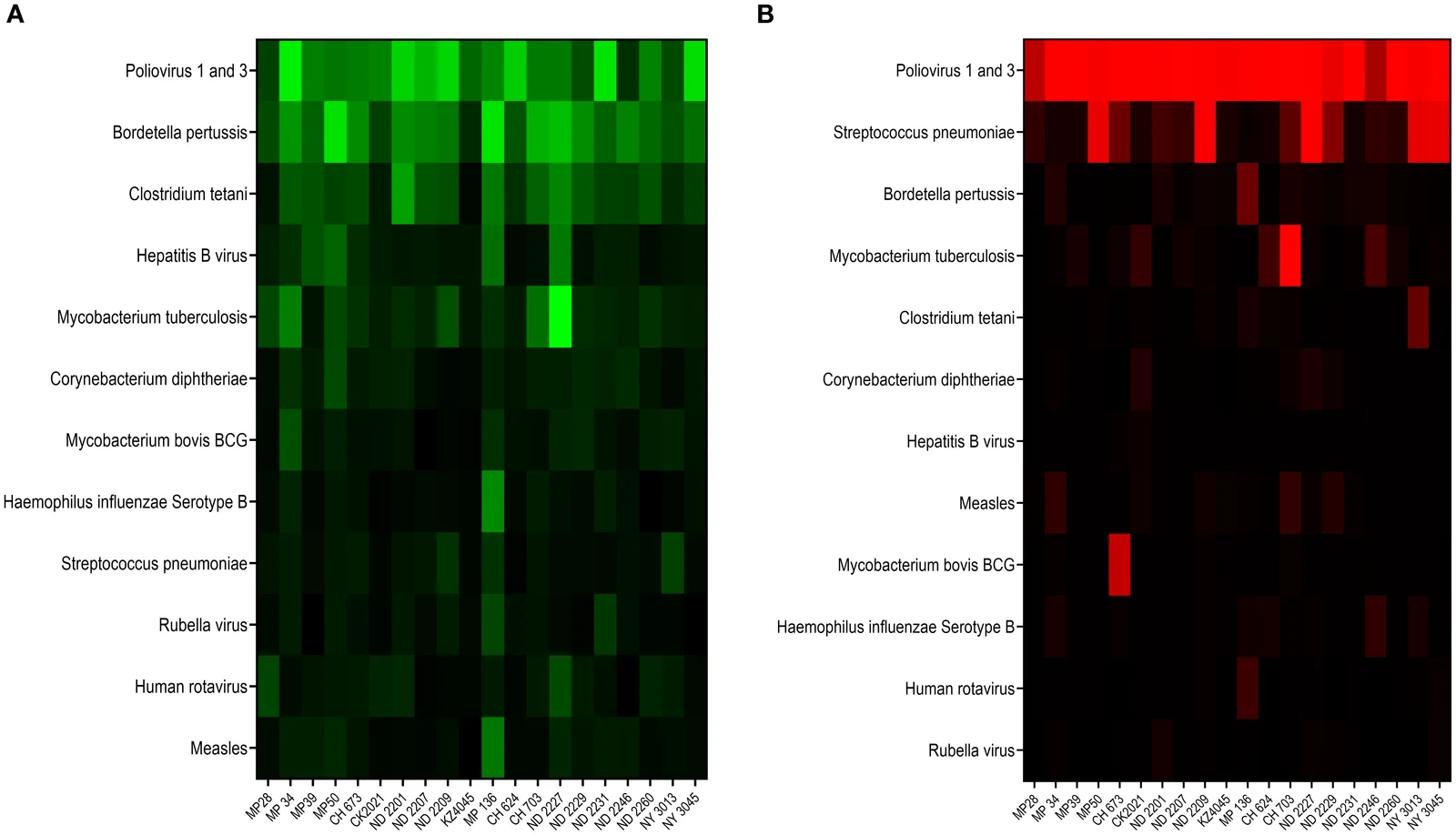
**(A, B)** Heatmaps of **(A)** IgM and **(B)** IgG reactivities of each sample to a panel of childhood vaccine pathogens. The vaccine antigens included in the bespoke microarray chip are along the y axis, and the 20 samples included in the study are reflected on the x axis. IgM reactivities are expressed in green **(A)**, and IgG reactivities are expressed in red **(B)** with stronger reactivities appearing brighter.

### 3.4 Seroreactivity patterns of the sample population

The assay screened the reactivity of the children against 15-mer peptides from 280 pathogens, allergens, vaccines, autoantigen immunogens. The assays revealed complex and heterogenous IgM and IgG response profiles presented as heat maps in **Figure 2**. Notably, enteroviruses elicited particularly strong IgG reactivity across samples.

**Figure 2:**
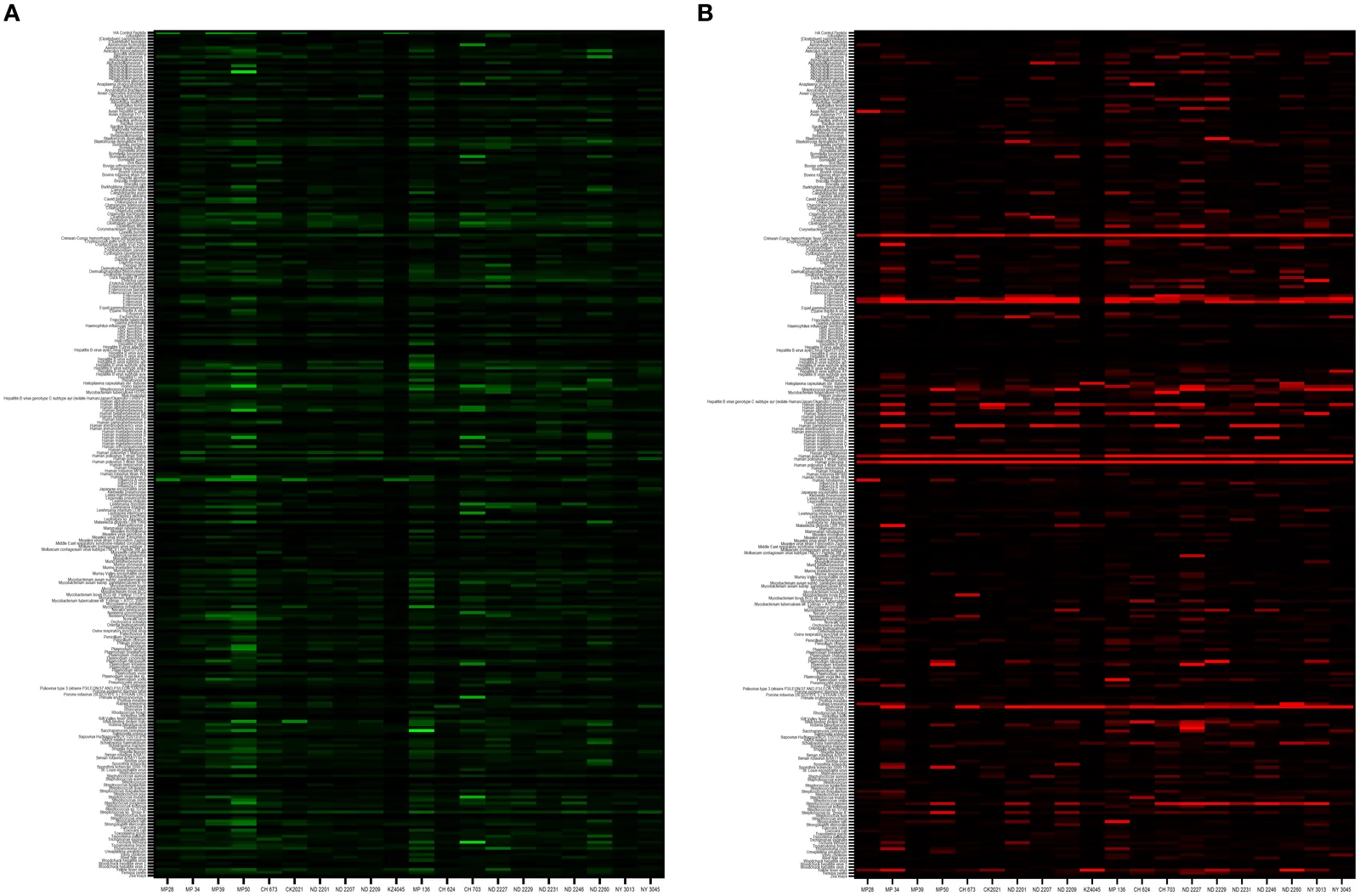
**(A, B)** Heatmaps of **(A)** IgM and **(B)** IgG reactivities of each sample to a panel of 280 immunogens. The 280 immunogens included in the bespoke microarray chip are shown along the y axis, and the 20 samples included in the study are shown along the x axis. IgM reactivities are expressed in green **(A)**, and IgG reactivities are expressed in red **(B)** with stronger reactivities appearing brighter.

### 3.5 Signatures of recent exposures in the population

Having established the ability of the microarray to detect known vaccine-associated antibody responses, we next examined IgM reactivity patterns across the broader panel of immunogens. As IgM is generally indicative of recent or ongoing antigen exposure, these responses provide insight into the immunogens currently shaping the immune landscape of this population. We determined antigen-specific IgM reactivities across the peptides on the microarrays to highlight current infection or recent exposure in the population. **Figure 3** illustrates the median IgM intensity levels for immunogens recognized by at least half of the study population. Autoantigens induced the highest reactivity across samples, followed by *Streptococcus* sp. Group A, Hepatitis B virus subtype adw 2, *Ehrlichia canis*, and *Streptococcus pneumoniae*. These immunogens were recognized by all 20 individuals in the sample population. Notably, Murray Valley Encephalitis, a virus endemic to Australia and Papua New Guinea, and *Trypanosoma cruzi*, a parasite endemic to the Americas, induced a relatively high IgM intensity across all 20 samples.

**Figure 3:**
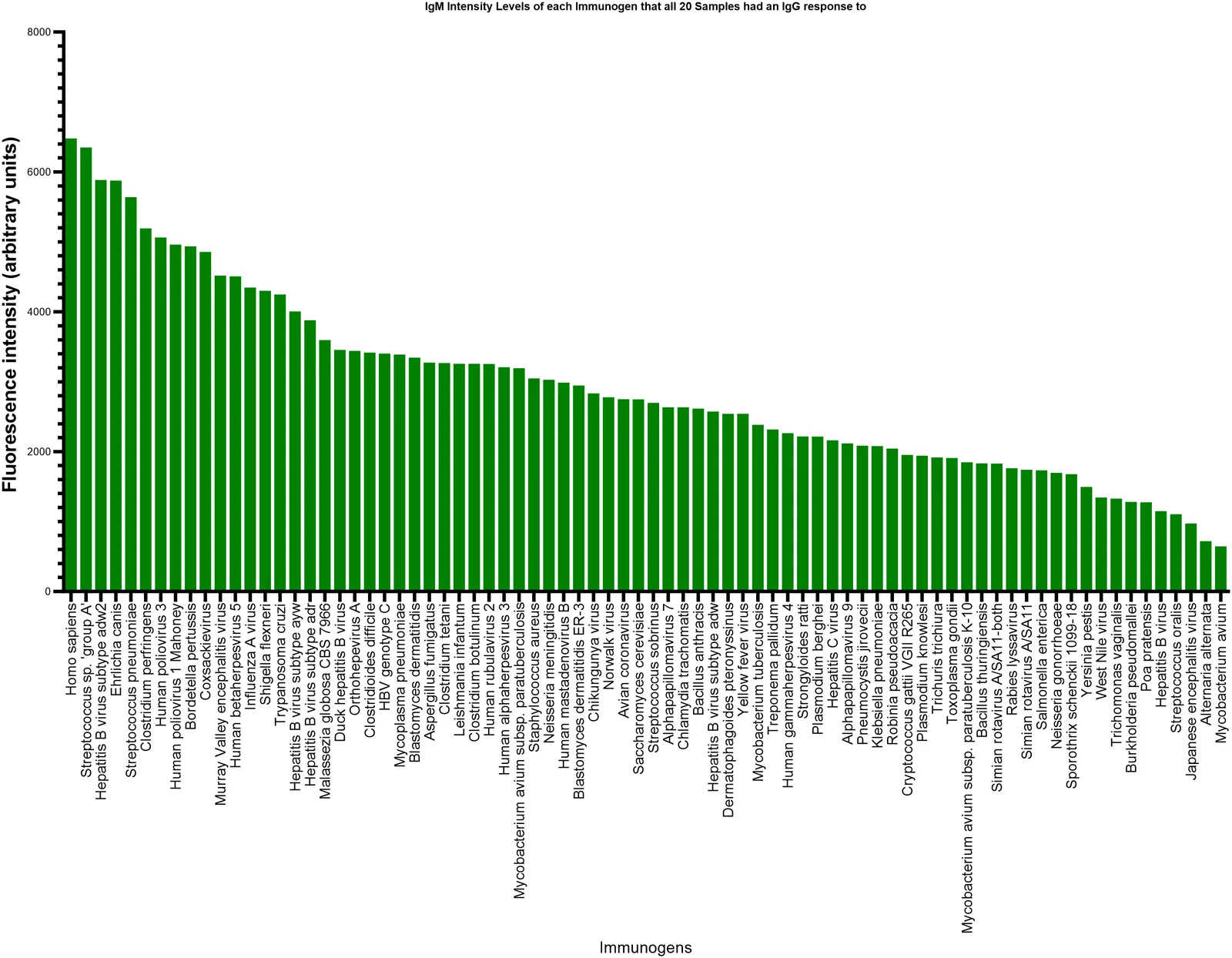
IgM intensity levels to the most population-relevant immunogens. This figure shows the intensity levels of immunogens that induced an antibody response in all 20 samples.

### 3.6 Historical exposures in the population

We also determined the antigen-specific IgG reactivities across the microarray peptides to identify historical exposures in the population. **Figure 4** illustrates the median IgG intensity levels to immunogens recognized by the entire study population. Human poliovirus 3 induced the highest IgG reactivity across samples, followed by human poliovirus 1 Mahoney, Coxsackievirus, Rhinovirus A, and Enterovirus C.

**Figure 4:**
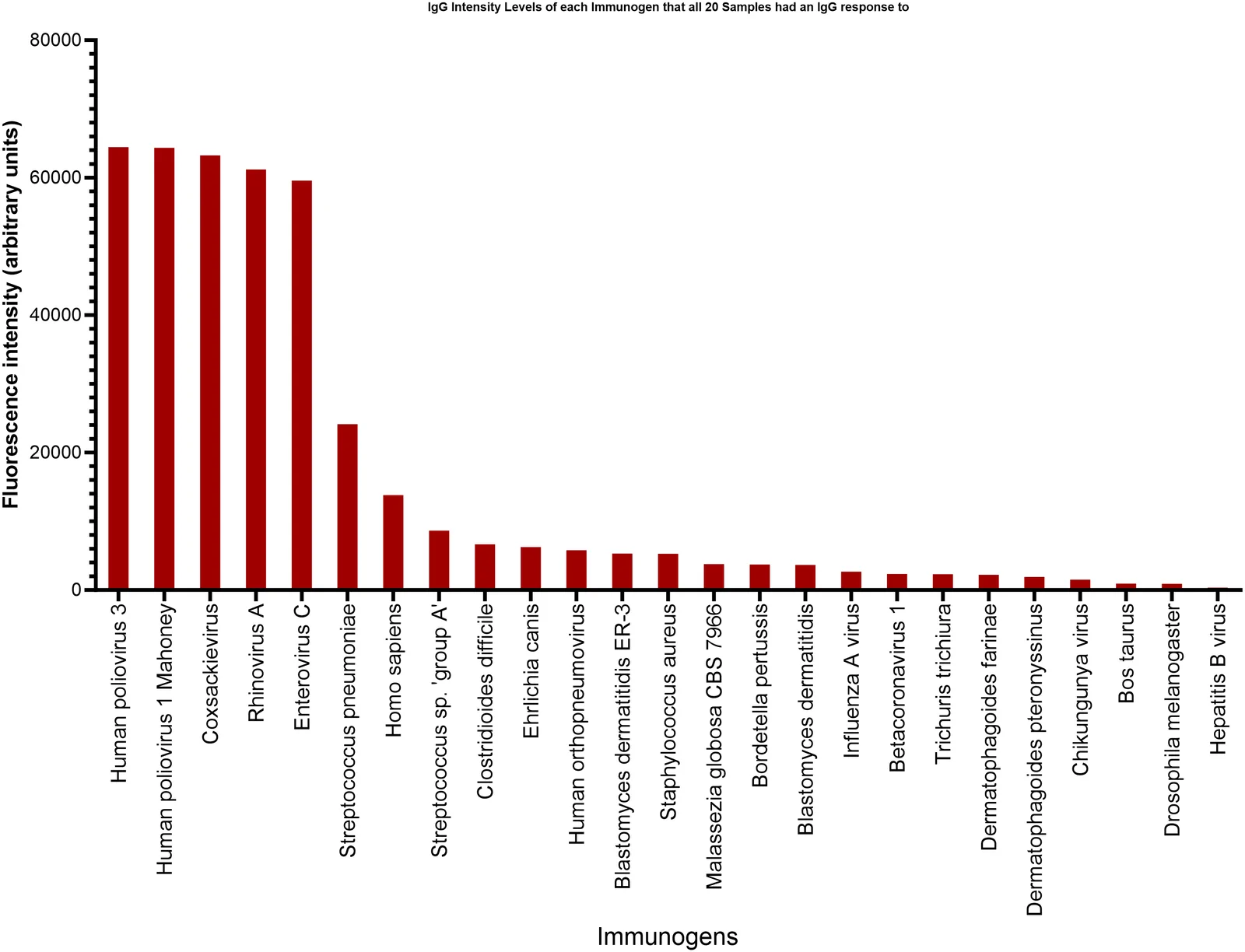
IgG intensity levels to the most population-relevant immunogens. This figure shows the intensity levels of immunogens that induced an antibody response in all 20 samples.

## 4 Discussion

### 4.1 Proof of concept and assay validation

The successful detection of antibody responses to known childhood vaccine antigens demonstrates that the bespoke peptide microarray can reliably identify expected antigenic exposures and provides confidence in its use for exposome mapping. We observed heterogeneous antibody responses to some vaccine antigens, with particularly low or absent reactivities to rotavirus and rubella in a subset of samples. Low or absent IgM responses were accompanied by detectable IgG, consistent with prior antigen exposure and class-switched immune responses. In other cases, low or absent reactivity may reflect incomplete vaccination histories. Such variability is well recognized in pediatric populations and may be influenced by age, genetics, nutritional status, concurrent exposures, and other host and environmental factors that affect vaccine immunogenicity and antibody kinetics (39). Temporal dynamics may further contribute, as systemic IgM peaks shortly after antigen exposure and IgG levels decline over time (40). Additionally, because peptide microarrays display linear epitopes, antibodies directed against conformational or structurally complex epitopes may not be detected, potentially underestimating responses to some antigens (41). Nevertheless, the overall detection of expected vaccine-associated antibody profiles demonstrates that high-throughput serological profiling can capture biologically meaningful exposure histories and provide insight into both vaccine-derived and naturally acquired immunity.

### 4.2 The childhood exposome is broad and heterogeneous

The extensive diversity and inter-individual variability of antibody responses observed in this cohort indicate that Zimbabwean children acquire highly heterogeneous antigenic exposure histories within the first five years of life. The extensive breadth of antigen recognition observed across individuals in this study is consistent with early, diverse exposure patterns reported in LMIC cohorts and contrasts with narrower antibody repertoires described in low-burden settings, where immune profiles tend to be less polyclonal in early childhood (30, 42–44). It is possible that the broad, heterogeneous infectome signature observed in this study may partially explain variations in clinical outcomes among children living in similar high-pathogen burden environments. This explanation is supported by the concept of immune imprinting, where early life antigen encounters leave lasting impacts on the response hierarchy and repertoire diversity (45, 46).

The breadth of immunogen recognition observed across the cohort highlights the wide range of antigenic exposures encountered in early life. Antibody responses were detected against diverse viral, bacterial, and autoantigens, reflecting a broad immunological exposome. Viral antigens, including vaccine schedule pathogens and enteroviruses elicited the highest IgG reactivity across samples. Strong IgM reactivity was observed against several non-viral immunogens, including autoantigens, *Streptococcus* sp. Group A, Hepatitis B virus subtype adw2, *Ehrlichia canis*, and *Streptococcus pneumoniae*, all of which were recognized by every individual in the cohort. The consistent recognition of these immunogens suggests common patterns of immune exposure arising from vaccination, environmental microbial contact, and circulating pathogens. Such early-life antigenic encounters may contribute to immunological imprinting, whereby prior exposures shape the developing immune repertoire and influence subsequent responses to infection, vaccination, and immune-mediated disease. This phenomenon of immunological imprinting has been shown for influenza virus, as early exposure shaped the B cell response to vaccination later in life (25).

The coexistence of strong IgM and IgG responses across multiple antigens within the cohort may suggest persistent and/or repeated antigen exposure and continuous immune stimulation. These patterns are consistent with previous observations that high pathogen-burden settings result in constant boosting and waning of humoral responses in children under 5 years old (26). Further, it aligns with prior research that demonstrates prolonged IgM persistence in pediatric populations exposed to endemic pathogens (47, 48).

### 4.3 IgM profile

The predominance of IgM responses to bacterial, viral and autoantigens suggests ongoing immune stimulation and highlights the dynamic nature of immune development during early childhood. IgM reactivity was strongest against autoantigens, followed by bacterial and viral antigens including *Streptococcus* spp. Group A, *Streptococcus pneumoniae*, hepatitis B virus subtype adw2, and *Ehrlichia canis*. These immunogens were recognized by all individuals in the study, suggesting either widespread recent exposure or sustained immune activation.

The predominance of auto-antigen-directed IgM responses is particularly notable and may reflect immune immaturity characteristic of early childhood, including incomplete central and peripheral tolerance mechanisms. Neonates and young children naturally express broad, low-affinity IgM repertoires prior to the full maturation of germinal center and class-switching pathways (22, 24, 49–51). Elevated natural IgM autoantibodies have been well described in neonates and young children and are thought to contribute to immune homeostasis, clearance of apoptotic cells, and early antimicrobial defense (50, 52, 53). Their prominence in this cohort underscores the importance of considering developmental immunology when interpreting pediatric serological profiles, particularly in high-exposure settings.

### 4.4 IgG profile

In contrast, IgG responses revealed a distinct antigenic hierarchy dominated by viral antigens, consistent with cumulative immune memory. The strong IgG responses to vaccine antigens and common childhood viruses reflect the establishment of immunological memory and provide evidence of cumulative antigenic experience early in life. Human poliovirus type 3 induced the strongest IgG reactivity across samples, followed by poliovirus type 1 (Mahoney strain), coxsackievirus, rhinovirus A, and enterovirus C. The prominence of poliovirus-specific IgG responses likely reflects early-life vaccination and/or natural exposure, consistent with the region’s widespread immunization programs (54). Strong IgG responses to multiple enteroviruses further suggest substantial viral circulation during early childhood, aligning with studies demonstrating frequent enteroviral exposure among children in LMICs (55). Seroepidemiological studies of enteric and respiratory pathogens across low-resource settings consistently show high IgG seroprevalence in young children, reflecting frequent early exposures (26). Collectively, these IgG-dominated profiles indicate that established memory to common viral antigens emerges early in life even amidst concurrent immune stimulation from other exposures.

### 4.5 Unexpected findings and cross-reactivity

Unexpected antibody reactivities to non-endemic pathogens likely reflect cross-reactive recognition of conserved epitopes, highlighting both the breadth and interpretative challenges of high-dimensional serological profiling. For example, a striking and unexpected finding was the relatively high IgM reactivity to Murray Valley encephalitis (MVE) virus across all samples, despite the virus being primarily endemic in Australia and Papua New Guinea. This is likely due to cross reactivity with antigenically related flaviviruses circulating in southern Africa, such as dengue, yellow fever, or West Nile virus, or, to a yet unidentified pathogen circulating in the population (56, 57). This may also be the case with the relatively high IgM reactivity found to *Trypanosoma cruzi*. Cross-reactivity has been reported in pediatric serology studies, where broad IgM patterns can capture exposure histories to related antigen groups not readily distinguished without functional assays (58). While cross-reactivity can complicate pathogen-specific attribution, IgM responses may also capture recognition of conserved or shared epitopes, offering insight into broader viral exposure patterns and immunogenic viral epitopes that may not be apparent through single-pathogen assays (10, 59, 60).

Antibody binding detected by the peptide microarray should not necessarily be interpreted as evidence of previous exposure to, or infection with, the corresponding pathogen. Although peptide microarrays allow for highly sensitive and broad serological profiling, assay specificity may be limited when peptides contain conserved or homologous epitopes shared between related organisms. Consequently, reactivity to non-endemic organisms, such as Murray Valley encephalitis virus and *Trypanosoma cruzi*, may represent cross-reactive antibody binding rather than true pathogen-specific exposure. This is particularly relevant for IgM, which can exhibit broader and less affinity-matured antigen recognition than IgG. In the absence of confirmatory pathogen-specific serological or functional assays, these unexpected signals should therefore be interpreted as evidence of antibody recognition of the represented peptide epitopes rather than definitive evidence of infection. Future studies incorporating confirmatory assays, such as pathogen-specific ELISAs, competition assays, or neutralization assays, would help distinguish true pathogen-specific responses from cross-reactivity and establish the specificity of these microarray signals.

### 4.6 The role of endemic co-infections in shaping the exposome

Chronic exposure to helminths and other endemic infections may contribute to the observed antibody profiles by influencing immune activation, regulation, and the development of humoral immune memory. Chronic co-infections, particularly with parasitic helminths, are characteristic of Zimbabwe and likely shape observed immune profiles in this cohort. Parasitic helminths, including schistosomes, have well-documented immunomodulatory properties that can affect humoral host responses. In chronic infections, helminths have been shown to alter humoral immune responses via immune skewing (61, 62), polyclonal B-cell activation (63), and impaired development of long-lived antibody memory (62, 64).

Helminth infections can attenuate or skew vaccine-specific responses and impact bystander antibody dynamics through altered T helper cell polarization and enhanced regulatory cytokine environments (62, 65, 66). Chronic helminth exposure is well known to induce Th2 and regulatory responses characterized by IL‐4, IL‐10, and TGF‐β production, which dampen Th1‐driven pathways and can suppress responses to unrelated antigens and vaccines (62). Helminth‐associated immunoregulation has been associated with reduced class switching to high‐affinity IgG antibodies and maintenance of more persistent IgM and IgE isotypes in some settings (67–69). In this context, pronounced IgM responses to bacterial, viral, and autoantigens may partially reflect helminth-associated immune activation.

Conversely, the strong IgG responses to viral antigens such as poliovirus and enteroviruses suggest that vaccine- and virus-induced immune memory remains robust in many children. The coexistence of broad IgM reactivity alongside established IgG memory suggests that early co-infections shape, rather than suppress, early-life immune development.

### 4.7 The importance of exposome mapping in children

Characterizing the childhood exposome provides a framework for understanding how cumulative antigenic experiences shape immune phenotypes, vaccine responsiveness, and susceptibility to disease. Understanding the range of exposures experienced during childhood is fundamental to epidemiological research and public health surveillance.

Although this study focused on children under five years of age, this developmental window captures rapid immune transitions, including waning of maternally derived IgG, expansion of antigen-experienced B-cell repertoires, and evolving immune regulation. This age group therefore provides a biologically informative starting point for defining population-level immune landscapes prior to surveillance across older age groups.

Taken together, the divergent IgM and IgG profiles underscore the complementary nature of these antibody classes in defining the pediatric exposome. IgG responses predominantly reflect cumulative immune memory to common viral pathogens and vaccine antigens, whereas IgM responses capture ongoing immune activation, early-life immune architecture, and potentially cross-reactive or conserved epitope recognition. Simultaneous profiling of IgM and IgG therefore provides a more nuanced insight into exposure histories, immune development patterns, and helps improve the interpretation of immune correlates of protection, which are often derived from low-burden settings and may not translate directly to high-burden environments. Conventional serological reference ranges and interpretations are largely derived from low-pathogen environments and may inadequately capture immunological normality in children with sustained pathogen exposure (33, 70). High-dimensional antibody profiling could help contextualize elevated or atypical antibody responses among children in high-burden settings, distinguishing adaptive immune activation from immune deficiency or pathology. In this way, infectome-informed frameworks may ultimately support more accurate interpretation of pediatric serology and immune status in high-burden settings.

### 4.8 Advantages of exposome mapping compared with existing approaches

Unlike conventional exposure assessment methods, multiplex serological profiling provides an integrated record of antigenic experience and enables simultaneous investigation of hundreds of immunogens from small sample volumes. Current approaches for assessing exposure include questionnaires, environmental monitoring, microbiological diagnostics, molecular detection methods, and increasingly, multi-omic approaches such as metabolomics and microbiome profiling. While each provides valuable information, they capture different aspects of exposure and are associated with important limitations. Questionnaires are subject to recall bias and incomplete records, particularly in settings where vaccination and clinical histories are not consistently documented. Environmental monitoring can identify pathogens or contaminants present within a child’s surroundings but cannot determine whether exposure occurred. Molecular diagnostics such as PCR and pathogen sequencing are highly sensitive for detecting active infection but provide only a snapshot of infection status at the time of sampling. Metabolomics and microbiome analyses provide insight into recent environmental, dietary, and microbial exposures but do not directly capture cumulative infectious exposure. In contrast, serological profiling measures the adaptive immune memory generated following antigen encounter and therefore provides a biologically integrated record of previous exposures that may persist long after pathogen clearance.

Within this context, high-throughput serological profiling represents a particularly valuable tool for investigating the infectious exposome. By simultaneously measuring antibody responses to hundreds of pathogen-derived antigens, vaccines, autoantigens, and allergens, the present microarray extends beyond conventional serology, which is typically restricted to a small number of predefined targets. Rather than assessing exposure to individual pathogens in isolation, this approach generates a high-dimensional representation of cumulative immune experience, providing insight into the breadth, diversity, and structure of antigenic exposures encountered throughout early life. The ability to derive such information from a small volume of pediatric serum further highlights its utility for large-scale population studies and surveillance programs in high-burden settings.

### 4.9 Limitations and future directions

While this proof-of-concept study demonstrates the utility of exposome mapping, larger longitudinal studies incorporating complementary immunological and clinical data are required to fully understand the drivers and consequences of immune heterogeneity.

A key limitation of this study is the relatively small sample size (n = 20), reflecting its proof-of-concept design. The primary aim was to demonstrate the feasibility and utility of the bespoke peptide microarray approach for characterizing the immunological exposome rather than to provide definitive population-level estimates or test predefined hypotheses. Consequently, the study was not formally powered to detect associations between participant characteristics and antibody responses, and these analyses should therefore be considered exploratory. The small sample size may also limit the generalizability of the observed patterns to the wider population of preschool-aged children. Nevertheless, the breadth and heterogeneity of antibody reactivity detected across the cohort demonstrate the potential of this approach and provide a basis for hypothesis generation. Validation in larger, appropriately powered cohorts will be important to confirm these findings and determine the extent to which the observed immune profiles are representative of the wider population.

An important limitation concerns the interpretation of IgM reactivity in relation to vaccination history. Vaccine-induced IgM is generally associated with the early humoral response and is less persistent than IgG; consequently, vaccination records cannot be considered a robust reference standard for IgM positivity when substantial time has elapsed since vaccination. Thus, low IgM sensitivity in this study should not be interpreted as evidence of poor vaccine-induced immunity or poor assay performance. Rather, the IgM analysis provides an exploratory measure of antibody reactivity detectable at the time of sampling, which may reflect recent antigen exposure, persistent or polyreactive IgM, or cross-reactive recognition of related antigens. In contrast, IgG reactivity is more appropriate for evaluating concordance with historical vaccination.

Other key limitations of this study include the inability to infer protective immunity or active infection from antibody reactivities alone and potential cross-reactivity. Microbiological confirmation, analyses of additional isotypes, and functional assays, which were not performed due to financial constraints, would further strengthen the results of this study. Expanding exposome analysis to a larger sample size and complementing with ELISA titrations would also strengthen the results of this study. As the bespoke microarray only included linear peptides, antibody responses to conformational epitopes or those with post-translational modifications are not captured in the current study. Additionally, longitudinal infectome profiling across all ages will be critical for defining immune development and maturation trajectories as well as temporal dynamics of exposure. Integration with clinical, microbiome, and parasitological data could bolster the ability of infectome signatures to predict vaccine responsiveness, identify and monitor therapeutic targets, and inform targeted public health interventions. Importantly, exposome profiling should be viewed as complementary to, rather than a replacement for, pathogen-specific diagnostics and clinical surveillance. Population-specific exposome data may also improve the interpretation of clinical serology and support the development of regionally relevant immune reference frameworks. At a broader level, the exposome provides a systems-level view of pathogen exposure that extends beyond single-pathogen serology and is particularly relevant in pediatric populations, where clinical diagnoses often underestimate true exposure burden.

### 4.10 Global health and equity implications

Finally, generating population-specific immune landscape data is both a scientific and equity imperative in global health research. Generating population-specific immune landscape data is essential for developing more representative immunology, improving intervention design, and ensuring that vaccines and public health strategies are informed by the biological realities of historically underrepresented populations. Conventional correlates of protection and immunogenicity benchmarks are often derived from populations with low pathogen exposure and may not translate accurately to endemic contexts. For example, many modern vaccines are designed and optimized in populations with low baseline inflammation, minimal chronic immune activation, and predictable microbiomes, assumptions that may be violated in African immune ecologies (71–73). As a result, vaccines deployed in such contexts may function as population-level harm-reduction tools rather than immunologically optimized solutions, a trade-off often accepted under high-burden conditions. Establishing population-specific baseline immune landscapes, as demonstrated here, is therefore essential for informing clinical trial design, interpreting vaccine efficacy, and avoiding the exclusion of immune diversity that can undermine vaccine confidence. Failure to account for immunological diversity risks perpetuating reference standards and intervention strategies that are poorly aligned with the biological realities of the populations they are intended to serve.

Overall, this study provides an initial, proof-of-concept exposome reference for Zimbabwean children and demonstrates the potential of peptide microarrays to capture the immunological footprint of populations exposed to intense and diverse infectious pressures early in life.

## Data Availability

The original contributions presented in the study are included in the article/**Supplementary Material**. Further inquiries can be directed to the corresponding author.
